# Hormesis: A Brief Reply to an Advocate

**DOI:** 10.1289/ehp.0901681

**Published:** 2010-04

**Authors:** Paul Mushak

**Affiliations:** PB Associates, Durham, North Carolina, E-mail: pandbmushak@cs.com

In his commentary in *Environmental Health Perspectives*, Calabrese (2009) offered a number of responses to my critique of hormesis methodology (Mushak 2009). Here I will provide a counterpoint to that effort.

- Calabrese (2009) falsely asserted that I erred in calculations associated with entry and evaluatory criteria for hormesis frequency, specifically by choosing the wrong denominator for examining the proportion of entry candidates eventually found to be hormetic using the most conventional form of statistical significance. The choice of a denominator for these calculations depends on the question asked. My key question was, What proportions of 668 dose–response entry candidates from 20,285 original articles, using the three criteria identified by Calabrese and Baldwin (2001), partition into each of three hormesis categories? A total of 245 of the 668 candidate dose–responses (37%) had hormetic character, but only 74 of those (30%) were derived using the typical statistical significance test, yielding 11% overall.
- Calabrese (2009) mischaracterized my statements about the reliability of the two unvalidated selection criteria (Mushak 2009). My comments addressed applying criteria to screening large databases of publications for a putative new phenomenon. I was not concerned about routine uses of statistical forms for empirical data (e.g., analyses using 95% confidence intervals on independent means).
- Calabrese (2009) misunderstood my concerns about the two tallies of dosing points (1,089 and 1,791 points) from two of his previous studies (Calabrese and Baldwin 2001, 2003b). The still unanswered question is how the 871 (80% of 1,089) control-equivalent and threshold response–compatible dosing points reported by Calabrese and Baldwin (2001) are mathematically incorporated into a high preponderance of hormetic dosing points (to a 2.5:1 ratio) they reported later (Calabrese and Baldwin 2003b). I was not concerned about simple counts.
- Calabrese misinterpreted my concern about clustered distributions in entry candidates in the 20,285 articles. I was not referring to publications in which the same information is recapitulated in multiple articles, but whether serial publications that described a given experimental approach but tested different substances were included in the articles database. The clustering pattern, although important, remains unexplained.
- Calabrese stated that the use of entry and evaluation criteria had been validated for both sensitivity and specificity. The question here is whether entry and evaluation criteria that established the original sets of hormetic, false-positive, and false-negative values were validly derived.
- Calabrese (2009) misunderstood and misapplied my rationale for including single sub-NOAEL (no observed adverse effect level) dosing points in the original database. He stated that virtually all of the dosing points within the selected 664 dose responses had been identified previously (Calabrese and Baldwin 2003b). However, in my commentary (Mushak 2009), I clearly conveyed that this step itself had an inherent positive bias and that it is not surprising that hormetic responses outnumbered negative ones. Calabrese was incorrect that including single sub-NOAEL points from the 20,285 articles adds negative bias; rather, such inclusion offsets and corrects an inherent positive bias.
- Calabrese challenged my discussion of the National Cancer Institute (NCI) yeast data set, arguing that the Crump analysis noted in my commentary (Crump 2007) was not peer-reviewed [of course, neither was the rebuttal letter by Calabrese et al. (2007) peer-reviewed]. Calabrese missed the point: Which of two plausible alternatives better addresses the truth of hormesis being present in the NCI data set? Calabrese (2009) noted that Crump’s approach introduced 8-fold more variability into the control group statistics, accounting for lack of hormetic evidence. Thereby, he conceded that alleged hormesis in the NCI yeast data lies within the range of determinable control (i.e., nonhormetic) responses.
- Calabrese (2009) challenged my critique of an earlier article on the National Toxicology Program dose-ranging program (Calabrese and Baldwin 2003a). He asserted that all levels of evidence should combine to support the cumulative 31% hormesis frequency. I disagree that poor evidence is just as good as strong evidence; only their “moderate to high” and “high” evidence should have been used in their analysis, yeilding a combined 2.3% frequency and not the claimed 31%.The data of Calabrese and Baldwin (2003a) provided little meaningful support for 31% hormetic frequency.
- Calabrese (2009) objected to my discussing the language issues for hormesis; he argued that (hormesis) revisions are part of the nature of science and new phenomenology, and ignored my point that current hormesis definitions are either those of interpretive convenience or represent divergence rather than convergence (the usual path). One definition in my commentary (Mushak 2009) explained hormesis as an overcompensation for homeostatic preservation; the only discernible basis is as an explanation for U(J)-shaped or inverted U(J)-shaped curves. Another definition explained hormesis as three divergent phenomena.
- Calabrese (2009) took strong exception to my view that public agencies have been slow to address and accommodate hormesis within policy formulations. Regulatory agencies dealing with xenobiotics and human or ecologic health—the key issue—have not adopted hormesis.
